# A New Take on Prion Protein Dynamics in Cellular Trafficking

**DOI:** 10.3390/ijms21207763

**Published:** 2020-10-20

**Authors:** Rodrigo Nunes Alves, Rebeca Piatniczka Iglesia, Mariana Brandão Prado, Maria Isabel Melo Escobar, Jacqueline Marcia Boccacino, Camila Felix de Lima Fernandes, Bárbara Paranhos Coelho, Ailine Cibele Fortes, Marilene Hohmuth Lopes

**Affiliations:** Department of Cell and Developmental Biology, Institute of Biomedical Sciences, University of São Paulo, São Paulo, 05508-000, Brazil; rodrigo.nunes.alves@usp.br (R.N.A.); rebecapi@usp.br (R.P.I.); mariana.brandao.prado@usp.br (M.B.P.); isabelmelo@usp.br (M.I.M.E.); jacquelineboccacino@usp.br (J.M.B.); camila.felix@icb.usp.br (C.F.d.L.F.); coelhobarbarap@usp.br (B.P.C.); ailinesantosfortes@usp.br (A.C.F.)

**Keywords:** prion, PrP^C^, PrP, PrP^Sc^, vesicles, endocytosis, exosomes, trafficking

## Abstract

The mobility of cellular prion protein (PrP^C^) in specific cell membrane domains and among distinct cell compartments dictates its molecular interactions and directs its cell function. PrP^C^ works in concert with several partners to organize signaling platforms implicated in various cellular processes. The scaffold property of PrP^C^ is able to gather a molecular repertoire to create heterogeneous membrane domains that favor endocytic events. Dynamic trafficking of PrP^C^ through multiple pathways, in a well-orchestrated mechanism of intra and extracellular vesicular transport, defines its functional plasticity, and also assists the conversion and spreading of its infectious isoform associated with neurodegenerative diseases. In this review, we highlight how PrP^C^ traffics across intra- and extracellular compartments and the consequences of this dynamic transport in governing cell functions and contributing to prion disease pathogenesis.

## 1. Introduction

Cargo compartmentalization and transport—processes involving the formation of different types of transport vesicles within the cell for endocytic and exocytic trafficking—are essential for cell survival, signaling and homeostasis [1]. Cellular prion protein (PrP^C^) is a membrane-bound glycoprotein, preferentially located in lipid raft microdomains [2], where it interacts with different partners and organizes signaling platforms, modulating many molecular mechanisms [3,4]. Nevertheless, PrP^C^ is also capable of shifting dynamically throughout several cellular compartments, such as the cytosol, Golgi apparatus, endoplasmic reticulum (ER), and perinuclear region [5,6,7,8]. Additionally, after protein synthesis, processing and targeting to the cell surface, PrP^C^ can be internalized by different types of endocytosis, being primarily found in intracellular vesicles of several stages of the endocytic process [9,10]. The type of PrP^C^ internalization and the organelles involved in these mechanisms may trigger numerous signaling pathways and cellular outcomes [9].

Overall, the process of endocytosis involves the formation of protein-containing vesicles from distinct plasma membrane locations and, after internalization, different sets of cargoes can be routed to recycling or degradation [1]. Endocytosis is classified into clathrin-mediated and clathrin-independent processes [1]. In clathrin-mediated endocytosis (CME), clathrin acts as a heterodimer to form a lattice-like structure that coats the forming vesicle [11]. In addition, clathrin interacts with adaptor proteins, which in turn bind to phospholipids to assist the vesicle coating and the budding process [11]. Thereafter, the GTPase dynamin is recruited to the clathrin coat and forms a ring-shaped structure assisting vesicle fission along with myosin ATPases VI and 1E [12,13]. The large Ras superfamily of small GTPases (Rabs) is important during several steps of the endocytic process. These proteins often confer specificity to targeted vesicles during endocytosis, in addition to modulating the uptake of ligands and vesicle budding [14]. Rab5, for instance, assists the sequestering of ligands to the clathrin-coated pits and vesicle fusion to the early endosome [15]. Likewise, soluble N-ethylmaleimide-sensitive factor attachment protein receptor (SNARE) proteins are key molecules able to execute membrane fusion with the trafficking vesicle to ensure targeting specificity [16].

Other mechanisms of cargo internalization that do not involve clathrin are classified as clathrin-independent endocytosis. This process is responsible for the internalization of lipid raft-associated proteins, being highly sensitive to cholesterol depletion [17]. Among clathrin-independent mechanisms, the most common requires caveolin as a transmembrane scaffold protein, which binds to the cholesterol membrane and interacts with cytosolic coat proteins to form the caveolae, a flask-shaped oligomer that plays major roles in invagination [18].

Moreover, the heterogeneity of the plasma membrane regarding phosphoinositide metabolism, in addition to cholesterol and protein composition, can ultimately define the type of endocytic internalization [19]. After internalization, endocytic vesicles may progress through different mechanisms, being further sorted into two main routes: recycling to the plasma membrane or lysosomal/autophagic degradation, in which vesicles are identified by distinct protein compositions [14,20,21]. Rab11 is concentrated in recycling endosomes and presumably regulates the return of the cargo to the plasma membrane [14,22]. When the sequestered cargo is targeted to degradation, it undergoes the endocytic route with the assistance of Rab7 towards early endosomes, to late endosomes, and ultimately to lysosomes [14,22].

Once the sorting has occurred, intralumenal vesicles (ILVs) are formed upon invagination and there is subsequent detachment of specific portions from the endosomal membrane into its own lumen [23]. This process is highly dependent on the targeting of ubiquitinated molecules for lysosomal degradation, and ILVs accumulation culminates in the structuring of endosomal multivesicular bodies (MVBs) [23]. The fusion of MVBs with lysosomes will trigger degradation of the ILVs content by hydrolases [24]. Additionally, late endosome/MVBs can also fuse with plasma membrane and release the enclosed ILVs (then referred as exosomes) into the extracellular milieu [23]. Exosomes contain selective repertoires of biomolecules (nucleic acids, protein, lipids) able to orchestrate signaling pathways and trigger specialized functions in a variety of recipient cell types [25]. Formation and cargo sorting of ILVs/exosomes is mediated by a cooperative endosomal sorting complex required for transport (ESCRT) machinery that consist of four different protein complexes: ESCRT-0, -I, -II, -III and the associated AAA ATPase Vps4 complex (VPS4) [26].

It is now known that additional to traveling through distinct endocytic compartments, PrP^C^ can also reach the extracellular space via exosomes to play a plethora of functional roles according to its localization [27,28]. Moreover, PrP^C^ can be internalized by different mechanisms, including clathrin-, lipid-raft-, caveolin- and metal-dependent mechanisms [2,8,9,29]. In addition, membrane heterogeneity appears to influence the control of cargo uptake and destination [19]. In this sense, as a scaffolding protein, PrP^C^ binds with several distinct partners and may contribute to endocytic and exocytic trafficking dynamics [30].

Furthermore, post-translational misfolding of PrP^C^ can lead to its pathological form, called PrP^Sc^—a form associated with transmissible spongiform encephalopathies (TSE)—which benefits from cellular vesicle trafficking to convert PrP^C^ into its infectious isoform and propagate between cells [8,31,32]. PrP^C^ conversion to PrP^Sc^ occurs on the plasma membrane and continues after endocytic uptake, inside endosomal vesicles [8,10,33]. Therefore, substantial experimental evidence suggests that vesicular transport has an essential role in the physiological and pathological functions of the prion protein. In this review, we discuss the cellular trafficking of PrP^C^, new insights on the functional consequences of this dynamic transport, and how PrP^Sc^ takes advantage of these processes to convert PrP^C^ into the disease-associated form, spreading to other cells and tissues.

## 2. PrP^C^ Structure and Cellular Processing

Following its biosynthesis, PrP^C^ (or PrP) traffics dynamically through diverse membrane compartments to be processed, glycosylated, properly folded and then correctly anchored on the plasma membrane. PrP^C^ consists of two domains: The N-terminal, with four or five octapeptide repeats; and the globular C-terminal, composed of three α-helices and two antiparallel β-sheets [34]. The PrP^C^ gene, termed *PRNP*, encodes a 253-amino acid precursor protein, which is imported into the ER to be processed, glycosylated and folded into its final conformation before it traffics through the Golgi to the outer layer of the plasma membrane [35,36].

Nascent PrP^C^ is first translocated into the ER when the N-terminal signal sequence is recognized and bound by the signal recognition particles (SRP) and directed to the translocon [37]. Following its translocation, PrP^C^ loses its glycosylphosphatidylinositol (GPI) signal peptide on its C-terminal domain to receive a GPI anchor, resulting in the 208 amino acid mature protein [7,38]. The GPI anchor mediates the anchoring of PrP^C^ onto membranes.

**Figure 1:**
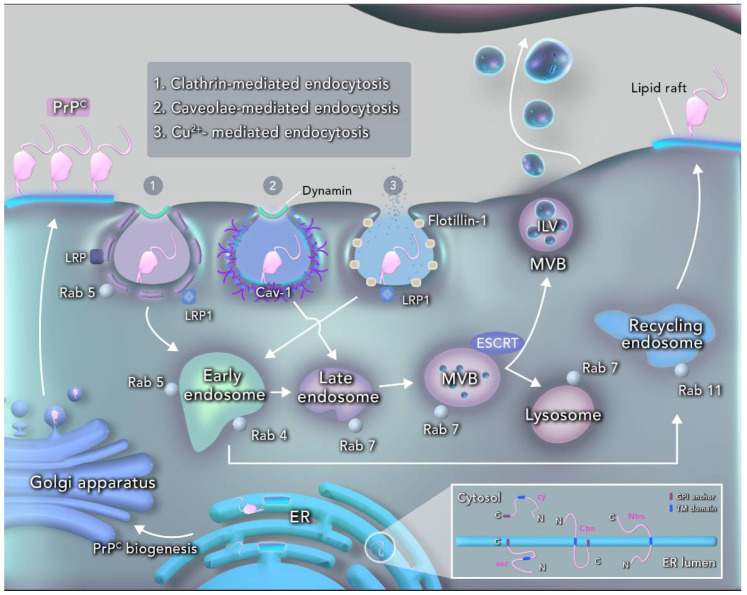
Biogenesis, location and endocytic trafficking of PrP^C^. PrP^C^ translocation to the ER may result in four different topological isoforms: ^sec^PrP, ^Ntm^PrP, ^Ctm^PrP and cyPrP (bottom right corner). ^sec^PrP is the most common isoform and is directed to the cell membrane after processing and maturation through the secretory pathway. PrP^C^ is preferentially located in lipid raft domains on the plasma membrane, where it can be internalized through clathrin-mediated (1) and clathrin-independent (2–3) endocytic pathways. (1) Clathrin-mediated endocytosis is the main route for PrP^C^ internalization, involving dynamin and the membrane receptors LRP and LRP1. (2) Caveolae-mediated endocytosis is a form of clathrin-independent endocytosis, where PrP^C^ interacts with dynamin and/or caveolin-1. (3) Another form of clathrin-independent internalization is Cu^2+^-mediated endocytosis of PrP^C^, which occurs due to its ability to bind to copper ions through the N-terminal domain, with the assistance of LRP1. Different endocytic compartments are identified by their specific proteins of the Rab GTPase family. As a result, of those processes, PrP^C^ can be sorted into the early (Rab5/Rab4/Rab6) or late endosomes (Rab7), as well as intraluminal vesicles (ILVs) in multivesicular bodies (MVBs) (Rab7), being targeted for recycling endosomes (Rab11), exosome secretion or lysosomal/autophagosomal degradation. Additionally, the ESCRT machinery assists PrP^C^ sorting into ILVs.

During its synthesis, PrP presents four topological isoforms according to its topological orientation in membranes: ^sec^PrP, ^Ctm^PrP, ^Ntm^PrP and cyPrP. ^sec^PrP, or secretory prion protein, is fully translocated into the ER lumen and it is the predominant isoform to be directed to the plasma membrane (Figure 1) [37]. However, in some instances, the translocation of the N-terminal domain stops, and an internal hydrophobic domain of PrP engages the translocon and generates the ^Ctm^PrP isoform, which spans the ER membrane once, with its N-terminal domain in the cytosol and its GPI anchor in the ER membrane [7,38]. ^Ntm^PrP also spans the membrane once, but has an opposite orientation compared with ^Ctm^PrP, with its N-terminal domain in the ER lumen and lacking a GPI anchor (Figure 1) [38]. The cellular functions of ^Ntm^PrP and ^Ctm^PrP are largely unknown, but a disruption in the proportion of these topologic isoforms has also been associated with neurodegeneration without accumulation of PrP^Sc^ [39,40]. Moreover, when there is a failure in the translocation of PrP^C^ to the ER, a cytosolic form of the protein (cyPrP^C^) is generated. This is generally due to an inefficiency or lack of the peptide signal. This isoform usually degrades rapidly, but the occurrence of cytosolic aggregates has been described during ER stress [41].

Furthermore, PrP^C^ can also be misfolded into the infectious form PrP^Sc^. Under healthy conditions, misfolded PrP is detected by the quality control system of the ER, triggering an intracellular signaling that ultimately leads to proteasomal degradation of the misfolded proteins. This degradation takes place in the ubiquitin-proteasome system (UPS) and occurs to approximately 10% of total synthetized PrP^C^ [42,43]. However, under ER stress or impairment of the UPS, PrP^C^ is prone to misfolding and forms cytosolic aggregates that are directly associated with prion-like diseases and neurodegenerative syndromes [44].

Continuing its processing, during transit from the ER to the Golgi, PrP^C^ undergoes N-linked glycosylation at Asn181 and Asn197, before being delivered to the cell surface [45]. PrP^C^ anchoring onto the plasma membrane depends heavily on the GPI domain and, like other GPI-anchored proteins, it is also found in specific microdomains called lipid rafts, that are enriched with cholesterol and sphingolipids [2,46]. PrP^C^ can undergo further post-translational modifications while in the plasma membrane, including proteolytic cleavages (namely the α-cleavage, β-cleavage and γ-cleavage) and shedding, which are critical regulatory alterations in both physiological and pathological events [47].

In sum, the biosynthesis of PrP^C^ and its domain architecture play a dominant role in the protein correct dispatch through the secretory pathway, and a failure in the PrP^C^ processing might contribute to the loss-of-function of the protein and/or accumulation of its pathological form, which will be discussed further in the next sections.

## 3. PrP^C^ in Intracellular Trafficking

GPI-anchored PrP^C^, preferentially located in lipid rafts on the cell membrane, functions as a cell surface receptor or co-receptor in concert with numerous ligands. PrP^C^ is transported by specific vesicular trafficking events through endocytic/secretory membrane systems to find a correct functional destination, and conversely towards recycling or degradation [48,49,50]. In different cell types, through distinct modes of internalization, and interacting with specific ligands, PrP^C^ triggers particular signaling cascades [9,51]. For instance, it was previously established that PrP^C^ was able to modulate p53-dependent cell death, increasing caspase 3 activity upon staurosporine induction, conferring a proapoptotic role to the protein [52]. Strikingly, as reported in mouse neurons and HEK293 cells, endocytosis of PrP^C^ is necessary for this proapoptotic activity, since specific mutations in the regions of the protein responsible for its internalization resulted in diminished p53-dependent caspase 3 activation-induced phenotype [53].

Additionally, the endocytosis of PrP^C^ is required for activation of Erk1/2 signaling by stress inducible protein 1 (STI1), a major PrP^C^ partner [51]. This study showed that, in hippocampal neurons, STI1-induced Erk1/2 activation was impaired in cells harboring PrP^C^-mutants devoid of endocytic activity [51]. Dynamin inhibition led to PrP^C^ and STI1 accumulation in the plasma membrane, suggesting that a dynamin-dependent internalization is essential for PrP^C^-STI1-Erk1/2 activation, modulating neuritogenesis [51]. In fact, the activation of MAPK and consequently Erk1/2 by signal transduction mediated by PrP^C^ can occur in several cell types, usually in lipid rafts [54,55,56]. However, it is not clear if or when PrP^C^ internalization is required for signaling initiation and propagation of most of the studied pathways. Nevertheless, further comprehension of how internalization occurs, and how membrane heterogeneity can impact this process may shed light on the physiological relevance of those events.

During its biosynthesis, PrP^C^ associates with lipid rafts in the ER to stabilize its conformation [57]. At the lipid raft, GPI-anchored proteins (GPI-APs) can be internalized despite the lack of intracellular sequences, usually associated with adaptor proteins at the membrane [9]. Lipid composition of membrane microdomains seems to control the endocytosis of GPI-APs, and those associated with fluid regions preferentially use early endosomes to be recycled, while GPI-APs associated with rigid domains are directed into the late endosome to be degraded [58]. Membrane heterogeneity regulates the entry of receptor and their cargoes into the endocytic pathways and it is decisive for each step of the sorting process [19]. Remarkably, the scaffold property of PrP^C^ may contribute to membrane heterogeneity through the formation of multi-protein domains in distinct locations of the plasma membrane and the surface of endosomes, favoring the recruitment of membrane remodeling proteins required for the endocytic journey.

CME, modulated by membrane heterogeneity at every step, is the main route for internalization of PrP^C^ (Figure 1). CME of PrP^C^ is mediated by its N-terminal portion and by adaptor proteins at the cell membrane [59,60]. The co-participation of the laminin receptor precursor (LRP) and the low-density lipoprotein receptor-related protein 1 (LRP1) has been reported, and both proteins are highly active in internalization processes of clathrin-coated pits, including in CME of PrP^C^ [59,60,61,62]. As such, the lack of clathrin and disruption of lipid rafts—by drugs such as filipin and nystatin [63] or inactivation of Cdc42, an actin-remodeling GTPase usually recruited to lipid rafts—blocks PrP^C^ internalization [49]. Association of PrP^C^ with lipid rafts remains during the whole CME process, suggesting that PrP^C^ and clathrin engage specifically at this location on cell membrane [49]. As briefly mentioned before, PrP^C^ internalization is dependent upon dynamin, and transits in endocytic vesicles positive for Rab5, prior to be accumulated in the perinuclear region [64]. Interestingly, Rab5A silencing leads to the impairment of PrP^C^ internalization and recycling to the cell surface [64].

PrP^C^ expression in caveolae is enriched, and clathrin-independent internalization of PrP^C^ may occur by caveolae [65]. PrP^C^ interacts with the transmembrane partners dynamin and/or caveolin-1, and caveolae containing internalized PrP^C^ are delivered to late endosomes and lysosomes, instead of recycling compartments [65] (Figure 1). Indeed, PrP^C^ is able to interact with caveolin-1 and phosphorylated Fyn at the octarepeat site of PrP^C^ [66]. Interestingly, PrP^C^ interaction with caveolin-1 regulates the activity of p59fyn in caveolae during neurite outgrowth [67] through the p42/44 MAP kinase/Erk1/2-signaling pathway [68]. It is likely that PrP^C^ is able to recruit its neuronal receptor NCAM to lipid rafts for p59fyn signaling activation and promotion of neuritogenesis [69]. In the absence of caveolin-1 and caveolae, endocytosis of PrP^C^ is preferentially mediated by clathrin [49,69,70].

In another form of clathrin-independent endocytosis, PrP^C^ can also be internalized by copper ions stimuli, functioning as a copper uptake molecule (Figure 1). PrP^C^ has the ability to bind copper ions through its flexible N-terminal domain. This specific binding seems to mediate structural changes in PrP^C^ moiety, stabilizing interactions between the N- and C-terminal regions, which may play a role as transporter and sensor of metal ions [71]. Interestingly, the aforementioned LRP1 also participates in PrP^C^ Cu^2+^-mediated endocytosis from lipid rafts in neuronal cells, when its octapeptide repeats domain is fully loaded with copper ions [61]. Strikingly, it was demonstrated that, in the presence of copper, flotillin-1 and PrP^C^ can engage and be internalized in a process dependent on the octarepeat domain of PrP^C^ [72]. It was also demonstrated that dynamin-1 plays a role in PrP^C^ internalization independently of copper stimuli, through a recycling pathway modulated by the small GTPase Arf6 [63].

PrP^C^ can be associated with ILVs from MVBs, being targeted to formation and secretion of exosomes, or for degradation through lysosome [73]. PrP^C^ is enriched in exosomes derived from a variety of cell types, including neural cells, and is also found in cerebral spinal fluid [74] and plasma [75]. Recent studies demonstrated that elevated levels of PrP^C^ in extracellular vesicles (EVs)/exosomes may play a role in intercellular signaling and it is implicated in early stages after stroke [76]. Interestingly, the levels of PrP^C^ in plasma exosomes of patients with Parkinson’s disease (PD) was directly correlated with cognitive impairment, suggesting a role for exosomal-PrP^C^ in the prognosis of PD [77]. Additionally, PrP^C^ is required for synaptic transmission, since its depletion considerably decreases the release of synaptic vesicles (SV) and affects the expression of several molecules related to vesicle recycling and fusion machinery, supporting the importance of PrP^C^ in exocytosis pathways [78].

The ESCRT machinery, involved in protein sorting to exosomes, is responsible for PrP^C^ selection as cargo during ILV formation [27] (Figure 1). The vacuolar protein sorting (VPS) components also participate in ESCRT mechanisms, modulating retrograde transport of proteins from endosomes to the trans-Golgi network [79]. Remarkably, inhibition of Vps35 expression leads to a decrease in the internalization rate of PrP^C^, while Vps28 silencing increases PrP^C^ endocytosis [80]. Moreover, dominant negative of Vps4 leads to enlargement of endosomes and accumulation of PrP^C^ [27]. Interestingly, overexpression of aberrant metabolic forms of PrP^C^ can block the fusion of autophagosomes with lysosomes, as well as their degradation capacity due to inactivation of Mahogunin Ring Finger-1 (MGRN1), an ubiquitin E3 ligase that also participates in ESCRT machinery [81].

Neutral sphingomyelinase 2 (nSMase2), an enzyme related to the generation of a bioactive lipid ceramide by hydrolysis of the membrane lipid sphingomyelin, can also modulate exosome biogenesis and PrP^C^ packaging, since nSMase2 knockdown impairs the association of PrP^C^ to exosomes [82].

Remarkably, recent data demonstrates that loss-of-PrP^C^ inhibits caveolin-1, which leads to formation of the Atg12-Atg5 complex, stimulating autophagy. In turn, increase of autophagy promotes degradation of MVB via lysosomes and reduction of exosome secretion [83]. Indeed, since we only started to understand the singularities involved in the secretory pathway, in-depth studies are needed to elucidate the molecular mechanisms involved in the sorting of PrP^C^ as cargo of exosomes and the functional role of the protein in these structures during their journey.

Thus, PrP^C^ traffics in different intra/extracellular compartments and across distinct domains in the cell membrane. The continued cycle of endocytosis and recycling prior to degradation may be important for modulating a cascade of signaling pathways that governs the cellular function of PrP^C^ (Figure 1). Recent data demonstrate that infectious prion strains can take advantage of distinct trafficking routes, that are, usually explored by PrP^C^, to spread. Therefore, the study of these processes is also crucial for the understanding of the pathogenesis and replication of prions and will be highlighted as follows.

## 4. Cellular Trafficking in PrP^Sc^ Infection

**Figure 2:**
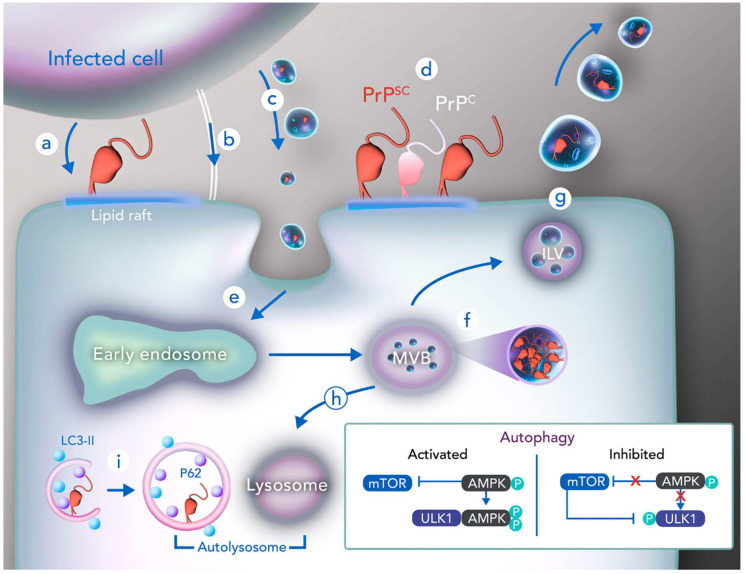
Prion infection mechanisms and PrP^Sc^ processing through autophagy. PrP^Sc^ is capable of catalyzing the conversion of PrP^C^ into the infectious isoform PrP^Sc^, and its spreading is highly dependent on PrP^C^ trafficking throughout different mechanisms, such as (**a**) prion infection through GPI-painting, in which PrP^Sc^ from an infected cell attaches to the lipid rafts of another cell; (**b**) prion infection through tunneling nanotubes, including transfer of endolysosomal vesicles in these structures; and (**c**) prion infection via exosomes, occurring in different cells types. It is postulated that PrP^c^-PrP^Sc^ conversion starts on the cell membrane (**d**) and continues throughout the endocytic trafficking (**e**–**g**). Once PrP^Sc^-containing exosomes fuse with the surface of other cell membrane (**e**), PrP^Sc^ can be directed to different endocytic vesicles. Multivesicular bodies (MVBs) can carry PrP^Sc^ to the autophagosomal/lysosomal degradation pathway (**h**), or to intraluminal vesicles (ILVs) to be secreted as exosomes (**g**). Under physiological conditions, mTOR is inhibited via AMPK phosphorylation, and PrP^Sc^ is sorted into the autophagosome (identified by markers p62 and LC3), being degraded after autophago-lysosomal fusion (forming the autolysosome) (inset: T bars represent pathway inhibition) (**i**). However, in prion-like diseases, mTOR inhibits the interaction between ULK1 and AMPK through phosphorylation of ULK1, leading to the impairment of autophagy and PrP^Sc^ clearance (bottom right corner).

As described above, PrP^C^ processing requires several post-translational modifications, which occur in distinct cellular compartments and depend on quality control performed by the ER [41,47]. Eventually, failures in the processing of PrP^C^ might lead to the aggregation and accumulation of misfolded prion proteins in the cytoplasm and a posterior advance of PrP^Sc^ agents (here, PrP^Sc^ or prion refers to disease-associated infectious form) to other cells [44]. PrP^Sc^ is capable of catalyzing the conversion of PrP^C^ to the infectious isoform (Figure 2), and it displays neurotoxic features, such as the capacity of accumulating in the brain [84] and, causing synaptic dysfunctions [85,86]. Pathological prion is related to the neurodegenerative effects of TSEs and also to the loss-of-function of physiological PrP^C^ [3]. Notably, while PrP^C^ is rich in α-helix, its infectious counterpart PrP^Sc^ is composed mainly of β-sheets [87]. As the transformation of one form to another consists of structure remodeling, it is possible to observe that the two isoforms are closely similar. PrP^C^-PrP^Sc^ conversion was demonstrated to take place in different circumstances, occurring during the intracellular traffic of PrP^C^ or when it is attached to lipid rafts on the cell surface (Figure 2) [88]. Interestingly, macropinocytosis was associated with PrP^C^ conversion to the infectious isoform in mouse neuroblastoma cells infected with PrP^Sc^ [89]. Furthermore, transformation of PrP^C^ into its pathological version may occur while PrP^C^ is associated with MVBs, since data from the literature describe that the impairment of MVBs maturation may decrease the rate of PrP^Sc^ infection [90]. Remarkably, the endosomal pH (~5.0) was demonstrated to be important for PrP^C^ conversion to the PrP^Sc^-like conformation [91].

Regarding its spreading, PrP^Sc^ has been proposed to disseminate among cells through distinct mechanisms, such as cell–cell contact [92], tunneling nanotubes (TNTs) [93] and exosomes [73,94].

First, cell–cell contact infection was determined by co-culture of PrP^Sc^-infected and healthy cells [92], and later the transmission of pathological prions from the peripheral to CNS was described, supporting cell–cell transmission [95]. This proposal of PrP^Sc^ spreading suggests that GPI-anchored PrP^Sc^ could be released from the lipid raft where it is located and attach to another raft, including one from a neighbor cell, in a process known as “GPI-painting” (Figure 2) [88]. Marshall et al. (2017) demonstrated that GPI-anchored PrP^C^ could be converted into a protease-resistant form of prion, [96], supporting this hypothesis. Additionally, the disruption of lipid rafts containing anchored PrP^C^ avoids the propagation of infectious prions [97]. On the other hand, it was demonstrated that non-anchored prions also present the ability to propagate the disease [98] with a slightly different phenotype, characterized by the absence of gray matter spongiosis and a slow widespread of amyloid depositions of PrP^Sc^ through the brain [99], forming plaques and neurofibrillary lesions [100], whereas anchored prions promote the deposition of non-amyloid PrP^Sc^ and extensive gray matter spongiosis [99].

In the second mechanism of spreading, PrP^Sc^ was demonstrated to be inside EVs secreted by distinct types of cells infected with pathological prions (Figure 2) [101]. Importantly, the intercellular transfer of PrP^Sc^ may occur through TNTs in endolysosomal vesicles, as demonstrated in a study with infected neuronal cells in mice [102]. In this work, it was also shown that the formation of TNTs augments in PrP^C^-overexpressing cells and neurons infected with pathological prions, and that PrP^Sc^ infection leads to higher membranous vesicle transferring, suggesting a promotion of infection mediated by both cellular and scrapie prions themselves [102].

Finally, data from the literature demonstrate how the inhibition of cholesterol trafficking and ceramide production [103], as well as the silencing of ESCRT machinery components [82], may lead to a decrease in exosome biogenesis and, consequently, to impairment of PrP^Sc^ spreading. In addition, it was reported that the presence of disease-associated prions leads to ER-stress associated apoptosis through negative modulation of MGRN1 [104]. On the other hand, vacuolation that causes spongiform-like encephalopathies provoked by prions has neuronal origins and it is independent of MGRN1 [105,106]. Misfolded prions with mutations at the C-terminal domain may not be recognized by ER quality control, reaching the Golgi apparatus and consequently the cell surface, as well as being internalized and carried by endosomes to lysosomes for degradation, with a fraction recycled back to the surface, maintaining the infection [107,108,109]. These findings highlight the importance of membrane dynamics and cellular trafficking for infectious prion pathogenicity. This is corroborated by other studies, which show that stimulating exosome secretion, through a drug named monensin, increases prion infection [110], and that isolated exosomes from prion-infected neural cells have the ability to originate new infections in healthy tissues and animals [94,111,112]. Additionally, since PrP^C^ may be present in lipid rafts that were incorporated into the membrane of exosomes, PrP^Sc^ is able to use this extracellular path to promote its own spread and disease progression [88].

Remarkably, studies with mouse neuronal cells show that infected exosomes contain both PrP^Sc^ and PrP^C^, although presence of PrP^Sc^ is related to some changes in these vesicles [113]. For instance, in contrast to non-infected exosomes, the infected ones show more divergent sizes and tend to have only one membrane layer in their structures [113]. When the PrP^Sc^-containing exosomes fuse with the surface of other cell, PrP^Sc^ is incorporated to it and becomes able to propagate the infection (Figure 2) [81,93]. Indeed, PrP^Sc^, in association with phospholipid vesicles (liposomes), presents a 10-fold increase in infectivity, and thus, vesicles play an essential role in prion infections [114]. Notably, PrP^Sc^ forms amyloid peptides which contain, in their structures, lipids derived from the membranes of vesicles that secrete other infectious prions [115].

Regarding PrP^Sc^ internalization, canonical PrP^C^ internalization through clathrin-coated pits or caveolae was expected, but data from literature presented controversial results [88]. In a Creutzfeldt–Jakob disease (CJD) model, no differences were observed in caveolin-1 expression in infected and healthy nervous tissue [116]. However, the blockage of both canonical endocytic pathways, clathrin- and caveolin-mediated, presented discrepant outcomes according to early or late stages of infection and different PrP^Sc^ variant strains [117], denoting that there are plenty of unknown mechanisms through which PrP^Sc^ may act. As the *PRNP* gene is known to show several distinct mutations [118], it would be relevant to ascertain whether these differences in endocytosis might occur due to these various types of genomic alterations. Interestingly, PrP^C^ mutants at the N-terminal site present a lack of copper-mediated endocytosis, which could lead to neuronal apoptosis and symptoms of prion diseases as if they were infected with PrP^Sc^, supporting the importance of the endocytic pathway for PrP^Sc^ cell–cell transmission [31]. Data from the literature demonstrate that mouse neurons infected with protease-resistant prions internalize the protein aggregates through vesicles, exclusively lysosomes and late endosomes, but not early endocytic or synaptic vesicles [119]. Moreover, protease-resistant prions accumulate in extremities of neurites, and the intercellular traffic was observed as independent of endogenous PrP^C^ [119].

Indeed, in murine neural cell lines, both PrP^C^ and PrP^Sc^ are located in vesicles, especially late endosomes. However, PrP^Sc^ was exclusively present in vesicles from CNS-expressing flotillin-1, found in late endosomal vesicles enriched with lipid rafts and GPI-anchored proteins [120,121], similar to β-amyloid proteins observed accumulating in endosomal vesicles from Alzheimer’s Disease (AD) patients [121]. In this work, it was also observed that PrP^Sc^ was not found in LYAAT1-positive lysosomes (Figure 2), but it was accumulated in LAMP1-positive late endosomes generating aggregates [121]. Strikingly, PrP^Sc^ accumulation leads to the lysosomal degradation of sortilin, an important molecule involved in the cellular sorting of PrP^C^ and PrP^Sc^ [122]. In this condition, the trafficking of both of these isoforms to late endosomes for lysosomal degradation becomes impaired, which facilitates the accumulation and propagation of the infection [122].

In addition, prion infection can affect Rab7, impairing its association with the membrane and consequently avoiding lysosomal degradation and maturation, which supports the spread of prions [123]. Other Rab proteins, such as Rab4 and Rab6a, participate in intracellular trafficking of PrP^C^ and present a role in its conversion to PrP^Sc^, since the modulation of expression of these proteins directly affects the formation of PrP^Sc^ and its accumulation in the ER [124]. Remarkably, prion infection can affect the traffic of proteins to the membrane in a post-Golgi vesicular pathway, including PrP^C^, insulin-like growth factor 1 (IGF-1) receptor, and attractin, with an accumulation of PrP^Sc^ in recycling endosomes, leading to severe malfunctions in neurons [125].

It is noteworthy that several miRNAs have been identified to be associated with prion-infected exosomes, some of which are identifiable in body fluids, presenting potential as biomarkers for prion disease [88,126,127,128].

Many efforts have been made to explore the identification of vesicles as a diagnosis and prognosis method for prion diseases, supporting the practical importance of studying the role of membrane trafficking in TSEs.

In summary, the aforementioned data reveal the relationship established between scrapie prions and components of both cellular trafficking and membrane dynamics, and how they affect each other. Furthermore, these components have direct impacts on the pathogenicity of PrP^Sc^ agents. For instance, decreased exosome biogenesis causes the infectivity of prions to be reduced [82,103], which could be a potential target for therapy against TSEs.

## 5. Prion and Autophagy

Autophagy is a lysosome-mediated pathway in which cells recycle cytosolic proteins and organelles as a survival response to nutrient deprivation. Autophagy is also important for turnover of cytoplasmic content and may have a role in neurodegenerative diseases. Notably, autophagy inhibition through gene silencing or specific drugs favors infection by PrP^Sc^ [129]. In mammals, the failure of autophagy induction leads to neurodegeneration: mice lacking Atg5, a protein involved in autophagic vesicle formation in the nervous system, present accumulation of abnormal intracellular proteins, leading to inclusion bodies in the cytoplasm of neurons and deficits in motor functions [130,131].

Induction of autophagy by lithium, rapamycin, calcineurin, sulforaphane or other small molecules can reduce the levels of PrP^Sc^ through different mechanisms and signaling pathways [97,132,133,134]. Studies have shown that metformin may reduce prion spreading and infection in the neuronal cell line CAD5 by enhancing autophagy via the AMP activated protein kinase (AMPK) [135]. As shown in Figure 2, AMPK activates ULK1 through serine phosphorylation when the cell is under starvation, promoting autophagy. However, if the cell is not under nutrient deprivation, mTOR actively inhibits ULK1 through the phosphorylation of another serine, disrupting the interaction of ULK1 with AMPK [136]. Likewise, histone deacetylase 6 (HDAC6), a protein involved with aggresome formation in neurodegenerative diseases, is also upregulated in prion infection and is suggested to increase autophagy through modulation of the PI3K-Akt-mTOR signaling pathway, resulting in neuronal protection against PrP^Sc^ [137].

Additionally, the autophagy adaptor protein p62 is upregulated in prion-infected cells, co-localizing with PrP^Sc^ aggregates when the UPS is inhibited [138]. This study also demonstrated that p62 is involved in the formation of prion-containing aggresomes, and that p62 reduced the levels of PrP^Sc^ in infected cells [138]. PrP^C^ is also able to control the autophagic flux of neurons through the activation of alpha7 nicotinic acetylcholine receptor (α7nAchR) [139]. This activation was shown to have a protective effect against prion infection via autophagy induction [139]. In scrapie models, there is a dysregulation in the expression of autophagy genes. In less affected areas of the infected brain, upregulation of LC3 proteins and expression of p62 indicates a role for autophagic machinery in PrP^Sc^ clearance [140].

Besides being important for PrP^Sc^ clearance, autophagy can modulate the release of prions by exosomes. Knockout of Atg5 or inhibition of autophagy with wortmannin increases exosomal prion release in murine neuronal cells [141]. In transport vesicles, PrP^C^ can associate with muskelin, which facilitates lysosomal degradation instead of exosomal release of PrP^C^ [141]. Corroborating this, the injection of PrP^Sc^ in mice knockout for muskelin facilitates prion disease onset [142].

In contrast, the octarepeat region of PrP^C^ is thought to play a role in autophagy downregulation. In *PRNP*^(-/-)^ neuronal cells, LC3-II increased, as well as accumulation of autophagosomes [143]. This activation of autophagy was reverted by introduction of PrP^C^, but not when the PrP^C^ lacked the octapeptide repeat region [143]. Furthermore, in astrocytes, a similar phenomenon was observed. Inhibition of autophagy in *PRNP*^(-/-)^ primary astrocytes re-established exosome release and the octapeptide repeat region of PrP^C^ was shown to be essential for this process [83]. Moreover, the octapeptide repeat region impaired the autophagosome formation induced by the Cav1-depedent Atg12-Atg5 complex in these astrocytes [83]. Therefore, further understanding of PrP^C^ regulation of autophagy versus exosome release is needed. This seemingly opposing effects of PrP^C^ and PrP^Sc^ in autophagy and exosome release could be context-dependent, relying on the position of PrP^C^ on the plasma membrane and its binding and endocytic partners. Nevertheless, the use of autophagy inducers is being evaluated for treatment of prion diseases.

Several autophagy inducers have been tested successfully against prion infection in vitro, but they lacked those effects in vivo [131]. Imatinib mesylate, an inhibitor of the tyrosine kinase c-Abl, was shown to activate autophagy in mammalian cells [144] and to reduce PrP^Sc^ loads in the spleen and spinal cord of prion-infected mice, prolonging their survival [145]. However, imatinib mesylate does not cross the blood-brain barrier (BBB) efficiently, which hindered its effects after prion neuroinvasion [145]. On the other hand, rapamycin, the classical mTOR inhibitor, was shown to prolong the survival of prion infected mice in combination with lithium [132] or alone [146], which indicates a potential of rapamycin for crossing the BBB and reaching the brain. Another mTOR inhibitor with similar mechanism of action to that of rapamycin is FK506 (tacrolimus), which also increased survival of prion-infected mice [147].

The use of drugs to induce autophagy and modulate the re-routing of prions for degradation instead of exosome release needs further investigation on the mechanisms of prion infection and spreading. Additionally, the stage of the disease during administration of such drugs might play a role in the observed effectiveness. Nevertheless, these studies shed light on the importance of a better understanding of the autophagy process in prion infected cells and how it affects prion degradation and spreading.

## 6. Conclusions and Perspectives

Briefly, we discuss the endocytosis of PrP^C^, which occurs through both clathrin-mediated and independent mechanisms [9]. PrP^C^ conversion to PrP^Sc^ takes place in the plasma membrane and continues after endocytic uptake, inside endosomal vesicles [33,148]. Indeed, vesicles enable a favoring environment for prion conversion due to the close proximity of the proteins, while the acidic pH facilitates the transition to β-sheet forms [33,148]. Furthermore, endosomes enriched with PrP^Sc^ can be recycled back to the plasma membrane or be directed to the Golgi, where it can interact with nascent PrP^C^ [149,150].

As mentioned before, membrane heterogeneity is essential for endocytosis and relies heavily on specific lipid and protein composition. Additionally, data suggest that such heterogeneity is maintained throughout the endocytic pathway [19]. As a scaffold protein, PrP^C^ is able to organize signaling platforms and, therefore, protein distribution in its surroundings. As such, membrane heterogeneity that is maintained during the endocytosis process must rely on organizing molecules such as PrP^C^. Indeed, when we look at the players involved in such heterogeneity, many are capable of engaging with PrP^C^, such as flotillin-1 [72] and clathrin [49]. Moreover, proteins of the Rab family can also be postulated to interact with PrP^C^, as Rab4, Rab6a and Rab7 seem to play an important role in regulating PrP^Sc^ infection [123].

Additional to its role in TSEs, PrP^C^ and its internalization might also be key to the propagation of α-synuclein (α-syn) aggregates, which are known to cause neurodegenerative diseases such as PD [151]. Indeed, data suggests that the internalization of α-syn is facilitated and modulated by the presence of PrP^C^, with its depletion impairing α-syn uptake [152]. Likewise, cells infected with PrP^Sc^ also present a decreased internalization of α-syn [152]. Interestingly, PrP^C^ can also act as a receptor for amyloid β, proteins involved in AD [153]. Therefore, PrP^C^ internalization is not only a key player in the propagation of TSE, but it also has an indispensable role in the promotion of other common neurodegenerative diseases. Thus, a better understanding of PrP^C^ and its internalization process may contribute to develop new treatments for a great variety of nervous system diseases.

As an attempt to eliminate the accumulation of PrP^Sc^, the cell targets PrP^Sc^ to lysosomal and ubiquitin-proteasome degradation as well as exocytosis [149,150]. PrP^Sc^, however, continues to accumulate in the cells due to the inefficacy of the above-mentioned processes to overcome the rate of prion conversion, leading to PrP^Sc^ spread and infection of other cells through exocytosis [149,150]. The use of intracellular antibodies could prove to be a powerful weapon in the combat against TSE, as studies are exploring specific PrP^C^-binding intrabodies that can promote the diversion of PrP^C^ vesicular traffic to proteasomal degradation, inhibiting PrP^Sc^ formation [154]. Another important mechanism against PrP^Sc^ spread is autophagy degradation, since it recognizes prion mutants that cause infection, and promotes the partial degradation of aggregates [132,133]. Notwithstanding, drugs related to autophagy induction have been broadly investigated as an approach to control and reduce the infection of prions [131,132,133,134,135].

Along with these findings, it is necessary to develop an understanding of the mechanisms for prion spreading throughout the organism, since the cellular and infectious isoforms present differences in cellular trafficking. Those differences rely on their distinct molecular constitutions that may affect the signaling for transport to specific compartments, requiring detailed studies to understand each isoform dynamics. On the other hand, it is noteworthy that the mechanism of PrP^C^ internalization remains elusive, because despite several studies describing the methods for endocytosis, how each pathway is triggered is still unknow and further investigation is required to unveil these processes. On this note, recent developments in technology allow for a greater exploration of vesicle content, which could help shed light into unexplored pathways [155]. Not only that, but the ability to modulate the fusing of membrane through optical traps allows for manipulation of vesicle components, which can in turn affect trafficking and membrane dynamics, therefore being of great therapeutic significance [156]. Indeed, many researchers are focusing in countering PrP^C^ conversion by disturbing its intracellular vesicular transport a means to combat TSE promotion and spreading [154,157], which can benefit immensely from such emerging technologies. As such, data suggest that the key to TSE prevention and treatment seems to lie in the better understanding of the cellular trafficking pathways related to PrP^Sc^ predecessor, PrP^C^.

## References

[1] Hinze C., Boucrot E. (2018). Endocytosis in proliferating, quiescent and terminally differentiated cells. J. Cell Sci..

[2] Taylor D.R., Hooper N.M. (2006). The prion protein and lipid rafts. Mol. Membr. Biol..

[3] Martins V.R., Beraldo F.H., Hajj G.N., Lopes M.H., Lee K.S., Prado M.A., Linden R. (2010). Prion protein: Orchestrating neurotrophic activities. Curr. Issues Mol. Biol..

[4] Santos T.G., Lopes M.H., Martins V.R. (2015). Targeting prion protein interactions in cancer. Prion.

[5] Harris D.A. (2003). Trafficking, turnover and membrane topology of PrP. Br. Med. Bull..

[6] Nikles D., Vana K., Gauczynski S., Knetsch H., Ludewigs H., Weiss S. (2008). Subcellular localization of prion proteins and the 37 kDa/67 kDa laminin receptor fused to fluorescent proteins. Biochim. Biophys. Acta Mol. Basis Dis..

[7] Godsave S.F., Peters P.J., Wille H. (2015). Subcellular distribution of the prion protein in sickness and in health. Virus Res..

[8] Vorberg I.M. (2019). All the same? The secret life of prion strains within their target cells. Viruses.

[9] Prado M.A.M., Alves-Silva J., Magalhães A.C., Prado V.F., Linden R., Martins V.R., Brentani R.R. (2004). PrPc on the road: Trafficking of the cellular prion protein. J. Neurochem..

[10] Marijanovic Z., Caputo A., Campana V., Zurzolo C. (2009). Identification of an intracellular site of prion conversion. PLoS Pathog..

[11] Kumari S., Mg S., Mayor S. (2010). Endocytosis unplugged: Multiple ways to enter the cell. Cell Res..

[12] Ungewickell E.J., Hinrichsen L. (2007). Endocytosis: Clathrin-mediated membrane budding. Curr. Opin. Cell Biol..

[13] Jimah J.R., Hinshaw J.E. (2019). Structural Insights into the Mechanism of Dynamin Superfamily Proteins. Trends Cell Biol..

[14] Langemeyer L., Fröhlich F., Ungermann C. (2018). Rab GTPase Function in Endosome and Lysosome Biogenesis. Trends Cell Biol..

[15] Rodman J.S., Wandinger-Ness A. (2000). Rab GTPases coordinate endocytosis. J. Cell Sci..

[16] Jahn R., Scheller R.H. (2006). SNAREs—Engines for membrane fusion. Nat. Rev. Mol. Cell Biol..

[17] Le Roy C., Wrana J.L. (2005). Clathrin- and non-clathrin-mediated endocytic regulation of cell signalling. Nat. Rev. Mol. Cell Biol..

[18] Elkin S.R., Lakoduk A.M., Schmid S.L. (2016). Endocytic pathways and endosomal trafficking: A primer. Wien. Med. Wochenschr..

[19] Redpath G.M.I., Betzler V.M., Rossatti P., Rossy J. (2020). Membrane Heterogeneity Controls Cellular Endocytic Trafficking. Front. Cell Dev. Biol..

[20] McNally K.E., Cullen P.J. (2018). Endosomal Retrieval of Cargo: Retromer Is Not Alone. Trends Cell Biol..

[21] Trivedi P.C., Bartlett J.J., Pulinilkunnil T. (2020). Lysosomal Biology and Function: Modern View of Cellular Debris Bin. Cells.

[22] Neefjes J., Jongsma M.M.L., Berlin I. (2017). Stop or Go? Endosome Positioning in the Establishment of Compartment Architecture, Dynamics, and Function. Trends Cell Biol..

[23] Piper R.C., Katzmann D.J. (2007). Biogenesis and function of multivesicular bodies. Annu. Rev. Cell Dev. Biol..

[24] Xu H., Ren D. (2015). Lysosomal physiology. Annu. Rev. Physiol..

[25] Meldolesi J. (2018). Exosomes and Ectosomes in Intercellular Communication. Curr. Biol..

[26] Hurley J.H. (2015). ESCRT s are everywhere. EMBO J..

[27] Porto-Carreiro I., Février B., Paquet S., Vilette D., Raposo G. (2005). Prions and exosomes: From PrPc trafficking to PrPsc propagation. Blood Cells Mol. Dis..

[28] Hartmann A., Muth C., Dabrowski O., Krasemann S., Glatzel M. (2017). Exosomes and the prion protein: More than one truth. Front. Neurosci..

[29] Hooper N.M., Taylor D.R., Watt N.T. (2008). Mechanism of the metal-mediated endocytosis of the prion protein. Biochem. Soc. Trans..

[30] Haucke V., Neher E., Sigrist S.J. (2011). Protein scaffolds in the coupling of synaptic exocytosis and endocytosis. Nat. Rev. Neurosci..

[31] Campana V., Sarnataro D., Zurzolo C. (2005). The highways and byways of prion protein trafficking. Trends Cell Biol..

[32] Baral P.K., Yin J., Aguzzi A., James M.N.G. (2019). Transition of the prion protein from a structured cellular form (PrPC) to the infectious scrapie agent (PrPSc). Protein Sci..

[33] Borchelt D.R., Taraboulos A., Prusiner S.B. (1992). Evidence for synthesis of scrapie prion proteins in the endocytic pathway. J. Biol. Chem..

[34] Riek R., Hornemann S., Wider G., Billeter M., Glockshuber R., Wüthrich K. (1996). NMR structure of the mouse prion protein domain PrP(121-231). Nature.

[35] Tatzelt J., Winklhofer K.F. (2004). Folding and misfolding of the prion protein in the secretory pathway. Amyloid.

[36] Acevedo-Morantes C.Y., Wille H. (2014). The structure of human prions: From biology to structural models—Considerations and pitfalls. Viruses.

[37] Heller U., Winklhofer K.F., Heske J., Reintjes A., Tatzel J. (2003). Post-translational import of the prion protein into the endoplasmic reticulum interferes with cell viability: A critical role for the putative transmembrane domain. J. Biol. Chem..

[38] Song Z.Q., Zhao D.M., Yang L.F. (2013). Metabolism of minor isoforms of prion proteins: Cytosolic prion protein and transmembrane prion protein. Neural Regen. Res..

[39] Stewart R.S., Piccardo P., Ghetti B., Harris D.A. (2005). Neurodegenerative illness in transgenic mice expressing a transmembrane form of the prion protein. J. Neurosci..

[40] Hegde R.S., Mastrianni J.A., Scott M.R., DeFea K.A., Tremblay P., Torchia M., DeArmond S.J., Prusiner S.B., Lingappa V.R. (1998). A transmembrane form of the prion protein in neurodegenerative disease. Science.

[41] Stewart R.S., Harris D.A. (2003). Mutational Analysis of Topological Determinants in Prion Protein (PrP) and Measurement of Transmembrane and Cytosolic PrP during Prion Infection. J. Biol. Chem..

[42] Gilch S., Nunziante M., Ertmer A., Wopfner F., Laszlo L., Schätzl H.M. (2004). Recognition of lumenal prion protein aggregates by post-ER quality control mechanisms is mediated by the preoctarepeat region of PrP. Traffic.

[43] Chakrabarti O., Hegde R.S. (2009). Functional Depletion of Mahogunin by Cytosolically Exposed Prion Protein Contributes to Neurodegeneration. Cell.

[44] Dubnikov T., Ben-Gedalya T., Reiner R., Hoepfner D., Cabral W.A., Marini J.C., Cohen E. (2016). PrP-containing aggresomes are cytosolic components of an ER quality control mechanism. J. Cell Sci..

[45] Castle A.R., Gill A.C. (2017). Physiological functions of the cellular prion protein. Front. Mol. Biosci..

[46] Wulf M.A., Senatore A., Aguzzi A. (2017). The biological function of the cellular prion protein: An update. BMC Biol..

[47] Linsenmeier L., Altmeppen H.C., Wetzel S., Mohammadi B., Saftig P., Glatzel M. (2017). Diverse functions of the prion protein—Does proteolytic processing hold the key?. Biochim. Biophys. Acta Mol. Cell Res..

[48] Sarnataro D., Pepe A., Zurzolo C. (2017). Cell Biology of Prion Protein. Prog. Mol. Biol. Transl. Sci..

[49] Sarnataro D., Caputo A., Casanova P., Puri C., Paladino S., Tivodar S.S., Campana V., Tacchetti C., Zurzolo C. (2009). Lipid rafts and clathrin cooperate in the internalization of PrPC in epithelial FRT cells. PLoS ONE.

[50] Taraboulos A., Raeber A.J., Borchelt D.R., Serban D., Prusiner S.B. (1992). Synthesis and trafficking of prion proteins in cultured cells. Mol. Biol. Cell.

[51] Caetano F.A., Lopes M.H., Hajj G.N.M., Machado C.F., Arantes C.P., Magalhães A.C., Vieira M.D.P.B., Américo T.A., Massensini A.R., Priola S.A. (2008). Endocytosis of prion protein is required for ERK1/2 signaling induced by stress-inducible protein 1. J. Neurosci..

[52] Paitel E., Alves da Costa C., Vilette D., Grassi J., Checler F. (2002). Overexpression of PrPc triggers caspase 3 activation: Potentiation by proteasome inhibitors and blockade by anti-PrP antibodies. J. Neurochem..

[53] Sunyach C., Checler F. (2005). Combined pharmacological, mutational and cell biology approaches indicate that p53-dependent caspase 3 activation triggered by cellular prion is dependent on its endocytosis. J. Neurochem..

[54] Lewis V., Hooper N.M. (2011). The role of lipid rafts in prion protein biology. Front. Biosci..

[55] Monnet C., Gavard J., Mège R.M., Sobel A. (2004). Clustering of cellular prion protein induces ERK1/2 and stathmin phosphorylation in GT1-7 neuronal cells. FEBS Lett..

[56] Hugel B., Martínez M.C., Kunzelmann C., Blättler T., Aguzzi A., Freyssinet J.M. (2004). Modulation of signal transduction through the cellular prion protein is linked to its incorporation in lipid rafts. Cell. Mol. Life Sci..

[57] Sarnataro D., Campana V., Paladino S., Stornaiuolo M., Nitsch L., Zurzolo C. (2004). PrPC association with lipid rafts in the early secretory pathway stabilizes its cellular conformation. Mol. Biol. Cell.

[58] Lakhan S.E., Sabharanjak S., De A. (2009). Endocytosis of glycosylphosphatidylinositol-anchored proteins. J. Biomed. Sci..

[59] Shyng S.L., Heuser J.E., Harris D.A. (1994). A glycolipid-anchored prion protein is endocytosed via clathrin-coated pits. J. Cell Biol..

[60] Sunyach C., Jen A., Deng J., Fitzgerald K.T., Frobert Y., Grassi J., McCaffrey M.W., Morris R. (2003). The mechanism of internalization of glycosylphosphatidylinositol-anchored prion protein. EMBO J..

[61] Taylor D.R., Hooper N.M. (2007). The low-density lipoprotein receptor-related protein 1 (LRP1) mediates the endocytosis of the cellular prion protein. Biochem. J..

[62] Gauczynski S., Nikles D., El-Gogo S., Papy-Garcia D., Rey C., Alban S., Barritault D., Lasmezas C.I., Weiss S. (2006). The 37-kDa/67-kDa laminin receptor acts as a receptor for infectious prions and is inhibited by polysulfated glycanes. J. Infect. Dis..

[63] Kang Y.S., Zhao X., Lovaas J., Eisenberg E., Greene L.E. (2009). Clathrin-independent internalization of normal cellular prion protein in neuroblastoma cells is associated with the Arf6 pathway. J. Cell Sci..

[64] Magalhães A.C., Silva J.A., Lee K.S., Martins V.R., Prado H.V.F., Ferguson S.S.G., Gomez M.V., Brentani R.R., Prado M.A.M. (2002). Endocytic intermediates involved with the intracellular trafficking of a fluorescent cellular prion protein. J. Biol. Chem..

[65] Peters P.J., Mironov A., Peretz D., Van Donselaar E., Leclerc E., Erpel S., DeArmond S.J., Burton D.R., Williamson R.A., Vey M. (2003). Trafficking of prion proteins through a caveolae-mediated endosomal pathway. J. Cell Biol..

[66] Shi Q., Jing Y.Y., Wang S.B., Chen C., Sun H., Xu Y., Gao C., Zhang J., Tian C., Guo Y. (2013). PrP octarepeats region determined the interaction with caveolin-1 and phosphorylation of caveolin-1 and Fyn. Med. Microbiol. Immunol..

[67] Pantera B., Bini C., Cirri P., Paoli P., Camici G., Manao G., Caselli A. (2009). PrPc activation induces neurite outgrowth and differentiation in PC12 cells: Role for caveolin-1 in the signal transduction pathway. J. Neurochem..

[68] Toni M., Spisni E., Griffoni C., Santi S., Riccio M., Lenaz P., Tomasi V. (2006). Cellular prion protein and caveolin-1 interaction in a neuronal cell line precedes Fyn/Erk 1/2 signal transduction. J. Biomed. Biotechnol..

[69] Santuccione A., Sytnyk V., Leshchyns’ka I., Schachner M. (2005). Prion protein recruits its neuronal receptor NCAM to lipid rafts to activate p59fyn and to enhance neurite outgrowth. J. Cell Biol..

[70] Galvan C., Camoletto P.G., Dotti C.G., Aguzzi A., Ledesma M.D. (2005). Proper axonal distribution of PrPC depends on cholesterol-sphingomyelin-enriched membrane domains and is developmentally regulated in hippocampal neurons. Mol. Cell. Neurosci..

[71] Salzano G., Giachin G., Legname G. (2019). Structural Consequences of Copper Binding to the Prion Protein. Cells.

[72] Ren K., Gao C., Zhang J., Wang K., Xu Y., Wang S.B., Wang H., Tian C., Shi Q., Dong X.P. (2013). Flotillin-1 mediates PrPC endocytosis in the cultured cells during Cu^2+^ stimulation through molecular interaction. Mol. Neurobiol..

[73] Fevrier B., Vilette D., Archer F., Loew D., Faigle W., Vidal M., Laude H., Raposo G. (2004). Cells release prions in association with exosomes. Proc. Natl. Acad. Sci. USA.

[74] Vella L.J., Greenwood D.L.V., Cappai R., Scheerlinck J.P.Y., Hill A.F. (2008). Enrichment of prion protein in exosomes derived from ovine cerebral spinal fluid. Vet. Immunol. Immunopathol..

[75] Ritchie A.J., Crawford D.M., Ferguson D.J.P., Burthem J., Roberts D.J. (2013). Normal prion protein is expressed on exosomes isolated from human plasma. Br. J. Haematol..

[76] Brenna S., Altmeppen H.C., Mohammadi B., Rissiek B., Schlink F., Ludewig P., Failla A.V., Schneider C., Glatzel M., Puig B. (2020). Brain-Derived Extracellular Vesicles are Highly Enriched in the Prion Protein and Its C1 Fragment: Relevance for Cellular Uptake and Implications in Stroke. CSHL.

[77] Leng B., Sun H., Zhao J., Liu Y., Shen T., Liu W., Liu X., Tan M., Li F., Zhang J. (2020). Plasma exosomal prion protein levels are correlated with cognitive decline in PD patients. Neurosci. Lett..

[78] Peggion C., Stella R., Chemello F., Massimino M.L., Arrigoni G., Cagnin S., Biancotto G., Franchin C., Sorgato M.C., Bertoli A. (2019). The Prion Protein Regulates Synaptic Transmission by Controlling the Expression of Proteins Key to Synaptic Vesicle Recycling and Exocytosis. Mol. Neurobiol..

[79] Horner D.S., Pasini M.E., Beltrame M., Mastrodonato V., Morelli E., Vaccari T. (2018). ESCRT genes and regulation of developmental signaling. Semin. Cell Dev. Biol..

[80] Ballmer B.A., Moos R., Liberali X.P., Pelkmans L., Hornemann S., Aguzzi A. (2017). Modifiers of prion protein biogenesis and recycling identified by a highly parallel endocytosis kinetics assay. J. Biol. Chem..

[81] Majumder P., Chakrabarti O. (2015). Mahogunin regulates fusion between amphisomes/MVBs and lysosomes via ubiquitination of TSG101. Cell Death Dis..

[82] Guo B.B., Bellingham S.A., Hill A.F. (2015). The neutral sphingomyelinase pathway regulates packaging of the prion protein into exosomes. J. Biol. Chem..

[83] Dias M.V.S., Teixeira B.L., Rodrigues B.R., Sinigaglia-Coimbra R., Porto-Carreiro I., Roffé M., Hajj G.N.M., Martins V.R. (2016). PRNP/prion protein regulates the secretion of exosomes modulating CAV1/caveolin-1-suppressed autophagy. Autophagy.

[84] Linden R., Martins V.R., Prado M.a.M., Cammarota M., Izquierdo I., Brentani R.R. (2008). Physiology of the prion protein. Physiol. Rev..

[85] Le N.T.T., Wu B., Harris D.A. (2019). Prion neurotoxicity. Brain Pathol..

[86] Mallucci G.R. (2009). Prion neurodegeneration: Starts and stops at the synapse. Prion.

[87] Pan K.M., Baldwin M., Nguyen J., Gasset M., Serban A., Groth D., Mehlhorn I., Huang Z., Fletterick R.J., Cohen F.E. (1993). Conversion of α-helices into β-sheets features in the formation of the scrapie prion proteins. Proc. Natl. Acad. Sci. USA.

[88] Cheng L., Zhao W., Hill A.F. (2018). Exosomes and their role in the intercellular trafficking of normal and disease associated prion proteins. Mol. Aspects Med..

[89] Wadia J.S., Schaller M., Williamson R.A., Dowdy S.F. (2008). Pathologic prion protein infects cells by lipid-raft dependent macropinocytosis. PLoS ONE.

[90] Yim Y.I., Park B.C., Yadavalli R., Zhao X., Eisenberg E., Greene L.E. (2015). The multivesicular body is the major internal site of prion conversion. J. Cell Sci..

[91] Van Der Kamp M.W., Daggett V. (2010). Influence of pH on the human prion protein: Insights into the early steps of misfolding. Biophys. J..

[92] Kanu N., Imokawa Y., Drechsel D.N., Williamson R.A., Birkett C.R., Bostock C.J., Brockes J.P. (2002). Transfer of scrapie prion infectivity by cell contact in culture. Curr. Biol..

[93] Gousset K., Schiff E., Langevin C., Marijanovic Z., Caputo A., Browman D.T., Chenouard N., de Chaumont F., Martino A., Enninga J. (2009). Prions hijack tunnelling nanotubes for intercellular spread. Nat. Cell Biol..

[94] Vella L.J., Sharples R.A., Lawson V.A., Masters C.L., Cappai R., Hill A.F. (2007). Packaging of prions into exosomes is associated with a novel pathway of PrP processing. J. Pathol..

[95] Glatzel M., Aguzzi A. (2000). PrP(C) expression in the peripheral nervous system is a determinant of prion neuroinvasion. J. Gen. Virol..

[96] Marshall K.E., Hughson A., Vascellari S., Priola S.A., Sakudo A., Onodera T., Baron G.S. (2017). PrP Knockout Cells Expressing Transmembrane PrP Resist Prion Infection. J. Virol..

[97] Taraboulos A., Scott M., Semenov A., Avraham D., Laszlo L., Prusiner S.B. (1995). Cholesterol depletion and modification of COOH-terminal targeting sequence of the prion protein inhibit formation of the scrapie isoform. J. Cell Biol..

[98] Rangel A., Race B., Klingeborn M., Striebel J., Chesebro B. (2014). Unusual cerebral vascular prion protein amyloid distribution in scrapie-infected transgenic mice expressing anchorless prion protein. Acta Neuropathol. Commun..

[99] Chesebro B., Race B., Meade-White K., LaCasse R., Race R., Klingeborn M., Striebel J., Dorward D., McGovern G., Jeffrey M. (2010). Fatal transmissible amyloid encephalopathy: A new type of prion disease associated with lack of prion protein membrane anchoring. PLoS Pathog..

[100] Jansen C., Parchi P., Capellari S., Vermeij A.J., Corrado P., Baas F., Strammiello R., Van Gool W.A., Van Swieten J.C., Rozemuller A.J.M. (2010). Prion protein amyloidosis with divergent phenotype associated with two novel nonsense mutations in PRNP. Acta Neuropathol..

[101] Vilette D., Courte J., Peyrin J.M., Coudert L., Schaeffer L., Andréoletti O., Leblanc P. (2018). Cellular mechanisms responsible for cell-to-cell spreading of prions. Cell. Mol. Life Sci..

[102] Zhu S., Victoria G.S., Marzo L., Ghosh R., Zurzolo C. (2015). Prion aggregates transfer through tunneling nanotubes in endocytic vesicles. Prion.

[103] Vilette D., Laulagnier K., Huor A., Alais S., Simoes S., Maryse R., Provansal M., Lehmann S., Andreoletti O., Schaeffer L. (2015). Efficient inhibition of infectious prions multiplication and release by targeting the exosomal pathway. Cell. Mol. Life Sci..

[104] Kaul Z., Chakrabarti O. (2017). Tumor susceptibility gene 101 regulates predisposition to apoptosis via ESCRT machinery accessory proteins. Mol. Biol. Cell.

[105] Walker W.P., Oehler A., Edinger A.L., Wagner K.U., Gunn T.M. (2016). Oligodendroglial deletion of ESCRT-I component TSG101 causes spongiform encephalopathy. Biol. Cell.

[106] Silvius D., Pitstick R., Ahn M., Meishery D., Oehler A., Barsh G.S., DeArmond S.J., Carlson G.A., Gunn T.M. (2013). Levels of the Mahogunin Ring Finger 1 E3 Ubiquitin Ligase Do Not Influence Prion Disease. PLoS ONE.

[107] Ashok A., Hegde R.S. (2009). Selective processing and metabolism of disease-causing mutant prion proteins. PLoS Pathog..

[108] Satpute-Krishnan P., Ajinkya M., Bhat S., Itakura E., Hegde R.S., Lippincott-Schwartz J. (2014). ER stress-induced clearance of misfolded GPI-anchored proteins via the secretory pathway. Cell.

[109] Goold R., McKinnon C., Rabbanian S., Collinge J., Schiavo G., Tabrizi S. (2013). Alternative fates of newly formed PrPSc upon prion conversion on the plasma membrane. J. Cell Sci..

[110] Guo B.B., Bellingham S.A., Hill A.F. (2016). Stimulating the release of exosomes increases the intercellular transfer of prions. J. Biol. Chem..

[111] Klöhn P.C., Castro-Seoane R., Collinge J. (2013). Exosome release from infected dendritic cells: A clue for a fast spread of prions in the periphery?. J. Infect..

[112] Cervenakova L., Saá P., Yakovleva O., Vasilyeva I., de Castro J., Brown P., Dodd R. (2016). Are prions transported by plasma exosomes?. Transfus. Apher. Sci..

[113] Coleman B.M., Hanssen E., Lawson V.A., Hill A.F. (2012). Prion-infected cells regulate the release of exosomes with distinct ultrastructural features. FASEB J..

[114] Gabizon R., McKinley M.P., Prusiner S.B. (1987). Purified prion proteins and scrapie infectivity copartition into liposomes. Proc. Natl. Acad. Sci. USA.

[115] Sun Y., Hung W.C., Lee M.T., Huang H.W. (2015). Membrane-mediated amyloid formation of PrP 106-126: A kinetic study. Biochim. Biophys. Acta Biomembr..

[116] Xiao X., Shen P., Wang Z., Dang J., Adornato A., Zou L.S., Dong Z., Yuan J., Feng J., Cui L. (2017). Characterization of physiochemical properties of caveolin-1 from normal and prion-infected human brains. Oncotarget.

[117] Fehlinger A., Wolf H., Hossinger A., Duernberger Y., Pleschka C., Riemschoss K., Liu S., Bester R., Paulsen L., Priola S.A. (2017). Prion strains depend on different endocytic routes for productive infection. Sci. Rep..

[118] Bagyinszky E., Van Giau V., Youn Y.C., An S.S.A., Kim S. (2018). Characterization of mutations in prnp (PRION) gene and their possible roles in neurodegenerative diseases. Neuropsychiatr. Dis. Treat..

[119] Magalhães A.C., Baron G.S., Lee K.S., Steele-Mortimer O., Dorward D., Prado M.A.M., Caughey B. (2005). Uptake and neuritic transport of scrapie prion protein coincident with infection of neuronal cells. J. Neurosci..

[120] Fivaz M., Vilbois F., Thurnheer S., Pasquali C., Abrami L., Bickel P.E., Parton R.G., Van der Goot F.G. (2002). Differential sorting and fate of endocytosed GPI-anchored proteins. EMBO J..

[121] Pimpinelli F., Lehmann S., Maridonneau-Parini I. (2005). The scrapie prion protein is present in flotillin-1-positive vesicles in central- but not peripheral-derived neuronal cell lines. Eur. J. Neurosci..

[122] Uchiyama K., Tomita M., Yano M., Chida J., Hara H., Das N.R., Nykjaer A., Sakaguchi S. (2017). Prions amplify through degradation of the VPS10P sorting receptor sortilin. PLoS Pathog..

[123] Shim S.Y., Karri S., Law S., Schatzl H.M., Gilch S. (2016). Prion infection impairs lysosomal degradation capacity by interfering with rab7 membrane attachment in neuronal cells. Sci. Rep..

[124] Béranger F., Mangé A., Goud B., Lehmann S. (2002). Stimulation of PrPC retrograde transport toward the endoplasmic reticulum increases accumulation of PrPSc in prion-infected cells. J. Biol. Chem..

[125] Uchiyama K., Muramatsu N., Yano M., Usui T., Miyata H., Sakaguchi S. (2013). Prions disturb post-Golgi trafficking of membrane proteins. Nat. Commun..

[126] Boese A.S., Saba R., Campbell K., Majer A., Medina S., Burton L., Booth T.F., Chong P., Westmacott G., Dutta S.M. (2016). MicroRNA abundance is altered in synaptoneurosomes during prion disease. Mol. Cell. Neurosci..

[127] Shah S.Z.A., Zhao D., Hussain T., Sabir N., Yang L. (2018). Regulation of MicroRNAs-mediated autophagic flux: A new regulatory avenue for neurodegenerative diseases with focus on prion diseases. Front. Aging Neurosci..

[128] Liu W., Bai X., Zhang A., Huang J., Xu S., Zhang J. (2019). Role of Exosomes in Central Nervous System Diseases. Front. Mol. Neurosci..

[129] Heiseke A., Aguib Y., Schatzl H.M. (2009). Autophagy, prion infection and their mutual interactions. Curr. Issues Mol. Biol..

[130] Hara T., Nakamura K., Matsui M., Yamamoto A., Nakahara Y., Suzuki-Migishima R., Yokoyama M., Mishima K., Saito I., Okano H. (2006). Suppression of basal autophagy in neural cells causes neurodegenerative disease in mice. Nature.

[131] Abdelaziz D.H., Abdulrahman B.A., Gilch S., Schatzl H.M. (2019). Autophagy pathways in the treatment of prion diseases. Curr. Opin. Pharmacol..

[132] Heiseke A., Aguib Y., Riemer C., Baier M., Schätzl H.M. (2009). Lithium induces clearance of protease resistant prion protein in prion-infected cells by induction of autophagy. J. Neurochem..

[133] Aguib Y., Heiseke A., Gilch S., Riemer C., Baier M., Schätzl H.M., Ertmer A. (2009). Autophagy induction by trehalose counteracts cellular prion infection. Autophagy.

[134] Lee J.H., Jeong J.K., Park S.Y. (2014). Sulforaphane-induced autophagy flux prevents prion protein-mediated neurotoxicity through AMPK pathway. Neuroscience.

[135] Abdelaziz D.H., Thapa S., Abdulrahman B., Vankuppeveld L., Schatzl H.M. (2020). Metformin reduces prion infection in neuronal cells by enhancing autophagy. Biochem. Biophys. Res. Commun..

[136] Kim J., Kundu M., Viollet B., Guan K.L. (2011). AMPK and mTOR regulate autophagy through direct phosphorylation of Ulk1. Nat. Cell Biol..

[137] Zhu T., Zhao D., Song Z., Yuan Z., Li C., Wang Y., Zhou X., Yin X., Hassan M.F., Yang L. (2016). HDAC6 alleviates prion peptide-mediated neuronal death via modulating PI3K-Akt-mTOR pathway. Neurobiol. Aging.

[138] Homma T., Ishibashi D., Nakagaki T., Satoh K., Sano K., Atarashi R., Nishida N. (2014). Increased expression of p62/SQSTM1 in prion diseases and its association with pathogenic prion protein. Sci. Rep..

[139] Jeong J.-K., Park S.-Y. (2015). Neuroprotective effect of cellular prion protein (PrPC) is related with activation of alpha7 nicotinic acetylcholine receptor (α7nAchR)-mediated autophagy flux. Oncotarget.

[140] López-Pérez Ó., Otero A., Filali H., Sanz-Rubio D., Toivonen J.M., Zaragoza P., Badiola J.J., Bolea R., Martín-Burriel I. (2019). Dysregulation of autophagy in the central nervous system of sheep naturally infected with classical scrapie. Sci. Rep..

[141] Abdulrahman B.A., Abdelaziz D.H., Schatzl H.M. (2018). Autophagy regulates exosomal release of prions in neuronal cells. J. Biol. Chem..

[142] Heisler F.F., Pechmann Y., Wieser I., Altmeppen H.C., Veenendaal L., Muhia M., Schweizer M., Glatzel M., Krasemann S., Kneussel M. (2018). Muskelin Coordinates PrPC Lysosome versus Exosome Targeting and Impacts Prion Disease Progression. Neuron.

[143] Oh J.M., Shin H.Y., Park S.J., Kim B.H., Choi J.K., Choi E.K., Carp R.I., Kim Y.S. (2008). The involvement of cellular prion protein in the autophagy pathway in neuronal cells. Mol. Cell. Neurosci..

[144] Ertmer A., Huber V., Gilch S., Yoshimori T., Erfle V., Duyster J., Elsässer H.P., Schäzl H.M. (2007). The anticancer drug imatinib induces cellular autophagy. Leukemia.

[145] Yun S.W., Ertmer A., Flechsig E., Gilch S., Riederer P., Gerlach M., Schätzl H., Klein M. (2007). The tyrosine kinase inhibitor imatinib mesylate delays prion neuroinvasion by inhibiting prion propagation in the periphery. J. Neurovirol..

[146] Cortes C.J., Qin K., Cook J., Solanki A., Mastrianni J.A. (2012). Rapamycin delays disease onset and prevents PrP plaque deposition in a mouse model of Gerstmann-Sträussler-Scheinker disease. J. Neurosci..

[147] Nakagaki T., Satoh K., Ishibashi D., Fuse T., Sano K., Kamatari Y.O., Kuwata K., Shigematsu K., Iwamaru Y., Takenouchi T. (2013). FK506 reduces abnormal prion protein through the activation of autolysosomal degradation and prolongs survival in prion-infected mice. Autophagy.

[148] Harris D.A. (1999). Cellular biology of prion diseases. Clin. Microbiol. Rev..

[149] Pankiewicz J.E., Sanchez S., Kirshenbaum K., Kascsak R.B., Kascsak R.J., Sadowski M.J. (2019). Anti-prion Protein Antibody 6D11 Restores Cellular Proteostasis of Prion Protein Through Disrupting Recycling Propagation of PrP Sc and Targeting PrP Sc for Lysosomal Degradation. Mol. Neurobiol..

[150] Goold R., McKinnon C., Tabrizi S.J. (2015). Prion degradation pathways: Potential for therapeutic intervention. Mol. Cell. Neurosci..

[151] Stefanis L. (2012). α-Synuclein in Parkinson’s disease. Cold Spring Harb. Perspect. Med..

[152] Aulić S., Masperone L., Narkiewicz J., Isopi E., Bistaffa E., Ambrosetti E., Pastore B., De Cecco E., Scaini D., Zago P. (2017). α-Synuclein Amyloids Hijack Prion Protein to Gain Cell Entry, Facilitate Cell-to-Cell Spreading and Block Prion Replication. Sci. Rep..

[153] Laurén J., Gimbel D.A., Nygaard H.B., Gilbert J.W., Strittmatter S.M. (2009). Cellular prion protein mediates impairment of synaptic plasticity by amyloid-Β oligomers. Nature.

[154] Filesi I., Cardinale A., Mattei S., Biocca S. (2007). Selective re-routing of prion protein to proteasomes and alteration of its vesicular secretion prevent PrPSc formation. J. Neurochem..

[155] Morani M., Mai T.D., Krupova Z., Defrenaix P., Multia E., Riekkola M.L., Taverna M. (2020). Electrokinetic characterization of extracellular vesicles with capillary electrophoresis: A new tool for their identification and quantification. Anal. Chim. Acta.

[156] Vivek A., Bolognesi G., Elani Y. (2020). Fusing artificial cell compartments and lipid domains using optical traps: A tool to modulate membrane composition and phase behaviour. Micromachines.

[157] Cardinale A., Filesi I., Vetrugno V., Pocchiari M., Sy M.S., Biocca S. (2005). Trapping prion protein in the endoplasmic reticulum impairs PrPC maturation and prevents PrPSc accumulation. J. Biol. Chem..

